# Lysine-specific modifications of p53: a matter of life and death?

**DOI:** 10.18632/oncotarget.1436

**Published:** 2013-10-08

**Authors:** Diana Marouco, Alexander V. Garabadgiu, Gerry Melino, Nikolai A. Barlev

**Affiliations:** ^1^ Department of Biochemistry, University of Leicester, Leicester, UK; ^2^ Molecular Pharmacology Laboratory, Saint-Petersburg Institute of Technology, Saint-Petersburg, Russia; ^3^ MRC Toxicology Unit, University of Leicester, Leicester, UK; ^4^ Faculty of Medicine, University of Rome “Tor Vergata”, Rome, Italy; ^5^ Gene Expression Laboratory, Institute of Cytology, Saint-Petersburg, Russia

**Keywords:** p53, post-translational modifications, lysine methylation, acetylation

## Abstract

Post-translational modifications provide a fine-tuned control of protein function(s) in the cell. The well-known tumour suppressor p53 is subject to many post-translational modifications, which alter its activity, localization and stability, thus ultimately modulating its response to various forms of genotoxic stress. In this review, we focus on the role of recently discovered lysine-specific modifications of p53, methylation and acetylation in particular, and their effects on p53 activity in damaged cells. We also discuss a possibility of mutual influence of covalent modifications in the p53 and histone proteins located in the vicinity of p53 binding sites in chromatin and propose important ramifications stemming from this hypothesis.

## INTRODUCTION

**Figure 1:**
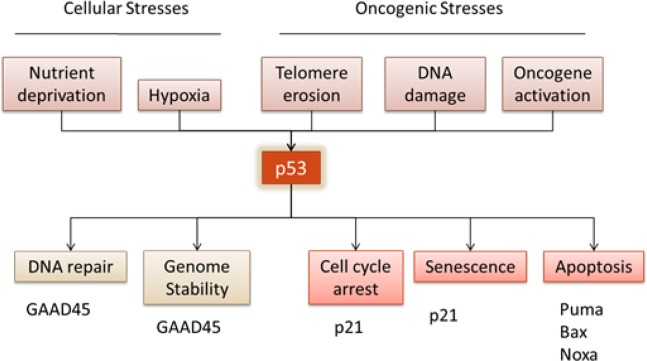
Tumour suppressor p53 regulates numerous cell responses. In response to cellular stresses, such as nutrient deprivation or hypoxia, or oncogenic stresses, such as telomere erosion, DNA damage or oncogene activation, p53 is activated triggering a wide range of signals.

p53 is a powerful transcription factor, that drives both the activation and repression of a large number of promoters, which ultimately define its tumour suppressor abilities [1,2]. The activation of p53 happens upon multiple stimuli, which can range from oncogenic stresses such as DNA damage [3] and genome instability [4], telomere erosion [5] and oncogene activation [6], to cellular stresses such as hypoxia [7] and re-oxygenation [8], or deregulation of cell metabolism due to nutrient deprivation [9-11]. As a consequence, p53 is responsible for the activation of many biochemical pathways, which result in different cellular outcomes, from DNA repair and temporary cell cycle arrest to apoptosis and senescence (Figure 1). In addition, although p53 exerts its function mainly as a sequence-specific transcription factor, it is also able to control various cellular processes via non-transcriptional mechanisms [12][13][14].

As such an important regulator, the control of p53 protein status becomes crucial for cancer progression [15,16]. However, the question of how p53 defines which programs of cell cycle arrest, DNA repair pathways, or apoptosis to initiate is still debatable. One mechanism for such specificity is likely mediated by the organization and composition of p53 binding sites in the regulatory regions of its target genes [17,18]. Another mechanism that accounts for the p53 specificity in execution of different cellular programmes (cell cycle arrest and DNA repair versus apoptosis) may be provided by a repertoire of stress-specific post-translational modifications (PTMs). Importantly, various PTMs affect both the p53 molecule and histones in the vicinity of its binding site(s) in chromatin, which together may orchestrate transactivation of target genes in a very precise manner [19].

**Figure 2:**
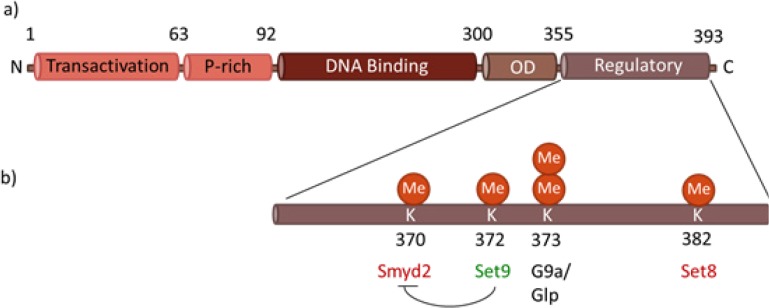
Functional domains of p53 a) Each monomer of the p53 protein contains four functional domains: the transactivation domain in the N-terminal region, followed by a proline-rich region, a DNA binding domain, an oligomerization domain (OD), and a C-terminal regulatory domain, where most of post-translational modifications occur. b) Methylation occurs at C-terminal lysines, within the regulatory domain of p53. Smyd2, Set9 and Set 8 catalyze the monomethylation of K370, K372 and K382, respectively, activating (green) or repressing (red) the activity of p53. G9a/Glp methylates K373, mainly resulting in dimethylation.

In terms of post-translational modifications, two regions of p53, the amino and carboxyl termini, are of special interest. The Transactivation domain (located in the N-terminus, residues 1-73) and the Regulatory domain (situated in the C-terminus, 360-393) are enriched with serines/threonines and lysines, respectively, which undergo PTMs mediated by various enzymes [20] (Figure 2a). PTMs affect the p53 protein by directly changing its physical properties and/or by forming new chemical surfaces for interactions with other proteins. According to the functional effects on p53, these PTMs can be divided into two groups: the ones that mark p53 for degradation and inactivation (ubiquitination, neddylation, sumoylation and methylation of certain lysines) and the ones that, on the contrary, stabilise and activate p53 (phosphorylation, acetylation, methylation of K372).

In this review, we focus on the functional importance of lysine-specific modifications that largely take place in the carboxyl terminus of p53, with a particular emphasis on methylation and acetylation. We also discuss how the interplay between these various modifications affects the function of p53 as a tumour suppressor and transcription factor. Finally, we propose that covalent modification(s) in p53 can serve as a prediction tool for searching the same modification in chromatin surrounding the p53 binding sites as well as the enzyme responsible for this modification, and vice versa.

### Acetylation of p53

#### P300/CBP HAT

The history of p53 acetylation goes back to 1997, when W. Gu and R. Roeder have found that the transcriptional co-activator p300/CBP (CREB binding protein), which was subsequently identified as a histone acetyltransferase (HAT), acetylated p53 both *in vitro* and *in vivo*[21]. Biochemical analysis uncovered that CBP had different specificity towards p53 *in vitro* and *in vivo*. p53 incubated with CBP *in vitro* underwent acetylation on multiple lysines in the carboxyl terminus (K370, K372, K373, K381, and K382) and yet *in vivo* the specificity of CBP was largely restricted to the two major acetylation sites, K373 and K382[21]. However, recent mass-spectrometry data argues that acetylation of all *in vitro* identified residues also takes place, and another CBP-acetylation dependent site in the DNA binding domain of p53 (K164) has been identified [22]. Notably, p300/CBP-mediated acetylation of p53 in cells was greatly enhanced by DNA damage and led to stabilisation of p53 on the protein level [21]. Importantly, although the abolishment of individual acetylation sites has no significant effect on p53 activity, the loss of all seven acetylation sites significantly decreases its ability to promote transcription, showing the redundancy of acetylation sites in p53 [22].

#### PCAF HAT

Another HAT, a P300/CBP-associated factor, PCAF, was shown to robustly acetylate p53 on K320 in the tetramerization domain on UV-induced DNA damage [3,23]. PCAF is part of a large multi-subunit transcriptional complex known as TFTC, or STAGA [24]. Surprisingly, acetylation of the K320 residue was shown to favour the survival of cancer cells in response to DNA damage insult. Apparently, this modification enhances the binding specificity of p53 for the promoter of p21 gene, thus halting cell cycle progression and allowing cells to repair [25]. These results were further corroborated by *in vivo* experiments using knock-in mice in which the TP53 mutant K317R (corresponds to K320R in humans) gene defective for PCAF-mediated acetylation has been introduced. Several types of tissues derived from the mutant animal, including thymocytes, epithelial cells from the small intestine and cells from the retina, exhibited a higher level of apoptosis after DNA damage, compared to the ones obtained from wild-type animals [26]. One plausible explanation to this effect may be that acetylation on K320 affects the ability of p53 to tetramerize properly, which is a pre-requisite for its successful binding to the low-affinity sites within the pro-apoptotic genes [27]. On the contrary, p21 (cell cycle arrest) and GADD45 (DNA repair) genes contain strong p53 binding sites, which allow p53 to bind these as dimers without a strict necessity for tetramerization [28]. More recently, PCAF-mediated acetylation was found to be mandatory for the maximal expression of p21, although this activity seems to be unrelated to p53 acetylation on K320, but rather is a consequence of histone acetylation in the p21 promoter [29].

#### Tip60 HAT

At present, in addition to CBP/p300 and PCAF, several other Histone Acetyltransferases (HATs) were shown to acetylate p53 in different structural regions. Tip60 (Tat-Interacting Protein 60) and MOF (Males absent on the first) are able to acetylate p53 in its DNA binding region on K120 [23,30]. Acetylation of this particular site occurs shortly after DNA damage, and seems to be an important mediator of p53-dependent apoptosis, without affecting cell cycle arrest [31].

#### Mechanisms of p53 activation by acetylation

While the positive role of acetylation in transcriptional activation by p53 is well defined, there is some controversy about the molecular mechanism of this phenomenon. On the one hand, it has been shown that a sharp upsurge of intracellular level of p53 facilitates the activation of its target genes. On the other hand, it is known that even in the absence of apparent stabilization, acetylation enhances p53-dependent transcription [22]. There are two plausible, yet not mutually exclusive explanations to this phenomenon. One possibility is that acetylation may facilitate the DNA-binding activity of p53, thus promoting the transcriptional activation of its target genes [21,32]. In line with this, the studies from W. Gu and S. McMahon groups support this hypothesis whereby acetylation may directly be involved in regulation of p53 DNA binding. Indeed, as mentioned earlier, Tip60 and MOF catalyse acetylation of p53 in the DNA binding domain (K120) augmenting the binding of p53 to promoters of pro-apoptotic genes [30,31]. In addition, a recent report shows that another acetyltransferase, MOZ (Monocytic leukemia zinc finger), is able to catalyse p53 acetylation on the same lysine residue (K120) resulting in its transcriptional activation [33].

Another possibility is that acetylation of p53, rather than enhancing the DNA binding activity of p53, promotes its interactions with various transcriptional HATs co-activators (e.g. p300/CBP, Gcn5 etc). The latter, in turn, modify local chromatin environment and facilitate the recruitment of RNA Polymerase II complex. Structural studies have shown that many HATs, including p300/CBP and PCAF, contain bromodomain, a specialized protein domain that recognises acetylated lysines. Subsequently, it has been shown that the C-terminally located K382 in p53 is recognised by the bromodomain of p300/CBP upon its acetylation by the latter [34,35]. Thus, the association between the p53 and p300/CBP proteins that initially has been formed via the amino-terminus of p53 and the KIX (CREB and MYB interaction) domain of p300/CBP would be further stabilised. As a result, the p53-p300/CBP complex becomes stably tethered to chromatin leading to acetylation of histones in the regulatory regions of p53-dependent genes [34,36]. The opposite scenario may also be true, whereby p300/CBP initially recruited to chromatin by p53, acetylates lysine residues in histones which provide further anchoring to the p300/CBP protein via the acetyl-lysine-bromodomain interaction. In line with this hypothesis is the fact that p300/CBP acetylates p53 more efficiently when the latter is bound to DNA [37], suggesting that this functional interaction occurs within the context of chromatin, rather than in the nucleoplasm.

The use of p53-mutant mice, in which acetylated lysine residues were mutated, shows a new insight in the importance of p53 acetylation in the modulation of its activity. For instance, although mice harbouring a mutation in K117 (corresponds to K120 in humans) displayed a complete abrogation of p53-mediated apoptosis, and loss of acetylation in all the p53 DNA-binding domain acetylation sites (K117, K161, K162) (p53^3KR^), prevents p53 from mediating cell cycle arrest, apoptosis and senescence *in vivo*, these mice are still not prone to early onset tumourigenesis [38]. These results suggest that loss of p53 classical responses – cell cycle arrest, apoptosis and senescence – are not critical for the tumour suppressor functions of p53. Moreover, p53^3KR^ mice maintain the ability to inhibit glycolysis and decrease the levels of ROS, showing that loss of acetylation does not interfere with other p53 functions such as metabolic regulation and anti-oxidant [38]. This report highlights the importance of less conventional functions of p53 for its tumour suppressor abilities, and emphasizes the role of acetylation in the modulation of p53 response. Another study using knock-in mice shows that p53 transactivation domain is essential for tumour suppression, although not by the activation of the conventional p53-target genes [39]. These findings unravel a new insight about p53 tumour suppressor abilities, distinct from cell-cycle arrest and apoptosis responses.

In addition, it should be noted that two recent studies have uncovered an important role of acetylation for the transcriptional-independent pro-apoptotic function of p53 [40,41]. Treatment of cells with an HDAC inhibitor (valproic acid) was shown to stabilize p53 acetylation on K120, and correlated with mitochondrial localization of p53, together with the pro-apoptotic protein BAX, thus triggering apoptosis [41]. These studies suggest a broader role of acetylation in the regulation of p53 activities.

#### Methylation of p53

In general, protein methylation occurs on the following amino acids: histidines, arginines, or lysines and is mediated by a special class of enzymes, called protein methyltransferases, which are structurally different from DNA methyltransferases. However, both types of methyltransferases use S-adenosyl-L-methionine (SAM) as a donor of methyl group [42]. In particular, lysine methylation is mediated by lysine methyltranferases (KMTases), which typically contain a SET (Su(var)3-9, Enhancer-of-zeste, Trithorax)[43] domain that facilitates the interaction between the methyl group and the target lysine to catalyse the methyl transfer reaction [44-48]. Lysine methyltransferases are able to mediate the transfer of up to three methyl groups to the ε-Nitrogen of the target lysine, thus generating either mono-, di- or tri-methylated lysines [49].

Until recently, methylation was regarded as a very stable and irreversible modification. The finding of the first lysine-specific demethylase (LSD1) by Shi and colleagues provided new insights regarding the reversibility of this modification, and revealed the dynamic nature of lysine methylation [50]. Following the discovery of LSD1, other proteins with histone demethylase activity were discovered, particularly a new family of proteins containing the JmjC domain [51].

The transfer of methyl groups to amino acids was first described to occur in histone proteins, contributing to the regulation of chromatin structure and function, and thus playing a crucial role in modulating gene expression [52-55]. More recently, however, other non-histone proteins were shown to undergo lysine methylation [56]. Among various substrates of lysine methylation, the tumour suppressor p53 is one of the most extensively studied, thereby providing a model for studying lysine methylation in non-histone proteins [56,57]. Lysine methylation of p53 occurs at least in four lysine residues, located in the C-terminal region of the protein: K370, K372, K373 and K382. Depending on both the location and on the extent of methylation state (mono, di or tri-methylation), p53 activity can either be enhanced or repressed [49,57](Figure 2b).

#### SET7/9 KMTase

Each KMTase possesses a high degree of substrate specificity, which is exemplified by the fact that four different KMTses methylate p53 specifically on each individual site. To date, there is only one methylation site, K372, which leads to activation and stabilisation of the p53 protein. K372 mono-methylation is mediated by Set7/9 KMTase (a.k.a. SetD7)[58]. This modification leads to the nuclear localization of p53, and enhances p53-dependent transcription of p21, BAX and MDM2 genes. In addition, p53-K372 methylation results in stabilization of the chromatin-associated p53 fraction [58]. These findings reveal a correlation between the methylation state of K372 and activation of p53 function. Furthermore, the level of methylated p53-K372 rapidly increased in response to DNA damage, whereas the levels of Set7/9 by itself remained largely unaffected. Collectively, these data indicate that stress conditions either quickly activate Set7/9 protein or enhance its recruitment to p53 [59].

Two recently published reports suggested that Set7/9 was dispensable for regulation of p53 activity in mice [60,61]. Both studies failed to observe methylation of the mouse version of p53 on K369 (corresponds to K372 in humans)[62]. This fact requires further investigation. On the one hand, several comparative studies on the role of post-translational modifications (phosphorylation and acetylation) of p53 in mouse and human cells yielded contradictory results, raising an important issue of inherent genetic differences between these model systems in respect to p53 regulation [63-65]. In addition, it should be noted that the majority of studies on p53 regulation by PTMs were carried out in cancer cell lines where the activity of p53 could be already compromised or altered. Therefore, ideally, the physiological effect of various PTMs on the activity of p53 should be investigated in matching non-transformed human cells.

#### SMYD2 KMTase

Methylation on three other target lysines leads to repression of p53. K370 is mono-methylated by KMTase Smyd2[66]. Smyd2 is a member of the SMYD (SET and MYND domain) family, which is characterized by the insertion of a MYND (myeloid-Nervy-DEAF-1) zinc finger within the SET domain.

Huang et al. [66] have shown that K370 methylation by Smyd2 resulted in transcriptional repression of p53 target genes, such as p21 and MDM2, due to the attenuation of p53 binding to the target promoters. Although very intriguing, this finding poses several challenging questions. For example, how such a small, neutrally charged modification, as lysine methylation, that takes place outside of the DNA binding domain, can attenuate the DNA binding ability of p53 in such a dramatic way? One plausible explanation to this is that mono-methylated K370 recruits a specific effector molecule that hinders the ability of p53 to bind DNA. Another possibility is that Smyd2, being recruited to chromatin, methylates not only p53, but also histones in the vicinity of p53-binding sites [67], thus inhibiting p53 binding. Future studies should help to resolve this nontrivial question. Furthermore, the level of K370 methylation was attenuated concomitant with the increase of K372 methylation in p53 after genotoxic stress [66]. This inhibitory effect of K372 methylation on K370 methylation is caused by the blockage of interaction between K370 of p53 and Smyd2, possibly due to the steric clash mediated by the neighbouring methyl group of K372 [68].

To further complicate this already complex picture, it was found that another, yet unknown methyltransferase is able to promote di-methylation of K370 [69]. This modification, contrary to the mono-methylation mark by Smyd2, activates p53 via facilitating its association with 53BP1 protein [69]. Importantly, this K370 di-methyl, but the not the mono-methyl mark, was specifically hydrolysed by LSD1 demethylase resulting in the inhibition of p53-dependent genes, including p21, MDM2 and PUMA. Since the level of p53-K370 di-methylation increases upon DNA damage, it is plausible that the activity/affinity of LSD1 should, in turn, diminish. Thus, it is tempting to speculate that the activity of LSD1 may be regulated by DNA damage-induced signal transduction pathway.

However, it must be noted that the effect of LSD1 on p53 is not as straightforward as it seems at first glance. In apparent contradiction to the data published by Huang et al.[69], Scoumanne and Chen [70] showed that LSD1 plays a positive role in p53 activation. Using several cell lines with inducible knockdown of LSD1, they demonstrated that LSD1 deficiency led to the delay of DNA-damage induced stabilization of p53, thus causing a slower induction of its target genes, p21 and MDM2 [70]. Importantly, the lack of LSD1 did not affect the expression of other p53 target genes, suggesting that this event is specific. One way to reconcile this apparent discrepancy is to assume that LSD1-mediated de-methylation of p53 is important to attenuate the binding of putative negative regulators of p53. Future experiments should shed light on this interesting question.

#### SET8 KMTase

Shi et al.[71] uncovered yet another p53-specific KMTase, Set8 (also known as PR-Set7 or KMT5a), that monomethylates p53 on K382. Similarly to Smyd2, Set8 inhibits p53 transcriptional activity. Methylation of K382 decreased the binding of p53 to the promoters of p21 and PUMA genes [71]. Also, the level of p53-K382me1 declined in response to DNA damage[71]. Later, it was found that K382me1 was the preferential binding site of the chromatin compaction factor L3MBTL1 thereby providing a molecular explanation to the inhibitory effect of K382 methylation on p53-dependent transcription [72]. Unexpectedly, an upsurge of p53-K382me1 binding was detected on the promoter of one of the p53-dependent DNA repair genes, GADD45, while it was attenuated on the promoters of other target genes involved in cell cycle arrest and apoptosis [71]. This result indicates that in tumour cells, K382 methylation may shift the p53-dependent transcription programme towards DNA repair. Therefore, K382me1 seems to have a more complex role in the regulation of p53 functions, other than unilateral repression of its target genes [71].

#### G9a/Glp KMTase

Recently, Huang and colleagues have reported that, in addition to acetylation and ubiquitylation, K373 of p53 can also be methylated by two homologous histone methyl- transferases, G9a and Glp [73]. The methylation level of K373 was not altered by DNA damage, which might suggest that di-methylation of K373 mediated by G9a/Glp correlates with inactive p53. In addition, simultaneous knockdown of G9a/Glp triggered apoptosis of tumour cells, further indicating that p53-K373me2 is a repressive mark [73]. However, is still unclear whether this cellular effect is a direct result of p53 K373 methylation, or whether it is caused by repressive methylation of histones in the promoters of pro-apoptotic target genes.

#### SETD2 and Smyd3 KMTases

In addition to KMTases that modulate p53 activity by direct methylation of the latter, several KMTases were shown to regulate p53 without its apparent methylation. For example, SETD2 was shown to directly interact with the N-terminus domain of p53 to selectively regulate its transcriptional activity [74]. This interaction leads to the up-regulation of many p53 target genes, including PUMA, NOXA, p53AIP1, Fas, p21, and Tsp1 [74]. However, SETD2 was unable to methylate p53 (N. B, unpublished work), signifying that the effects of SETD2 on the downstream targets of p53 are likely methylation-independent [74].

By analogy with Smyd2, another member of the SMYD family, Smyd3, which shares a high degree of similarity with KMTase Smyd2 [75], was also found interacting with p53 (N.B. and M. Rada, unpublished). Smyd3 was originally shown to methylate H3K4 [76], but more recently it has been proposed that H4K20 or H4K5 are likely the bona fide substrates of this enzyme [77,78]. Additionally, Smyd3 specifically methylates a non-histone protein, VEGFR1, leading to an up-regulation of its kinase activity [79]. Given that Smyd3 is overexpressed in several cancers including liver, breast and rectal carcinomas, it would be interesting to examine its potential role as a regulator of p53 activity.

### Functional outcomes of Interplay between Lysine Modifications in p53

#### Acetylation-Ubiquitylation interplay

**Figure 3:**
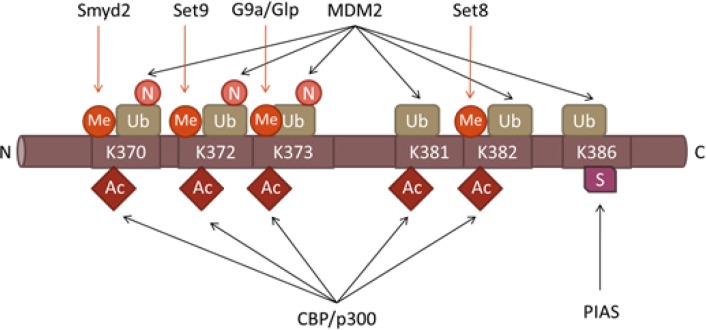
Post-translational modifications in the Carboxyl-terminal Domain of p53 Positions of various lysine-specific PTMS the C-terminal domain of p53 as well as the corresponding enzymes are indicated. The following abbreviations are used: Me-methylation, Ac-acetylation, Ub-ubiquitination, N-nedylation and S-sumoylation.

As evident from the previous section, methylation by itself ensures a significant degree of fine-tuning to p53 regulation. However, it is the interplay among different post-translational modifications that provides such a broad spectrum of possibilities for the control of p53 activities (Figure 3). In this respect, it is important to note that the C-terminal lysine residues of p53 can undergo different modifications: acetylation, methylation, ubiquitinylation and sumoylation, resulting in different functional outcomes.

Under normal conditions, p53 undergoes ubiquitylation by E3-specific ligases, Mdm2/Mdm4, which target the C-terminal lysines (K370, K372, K373, K381 and K382) in p53 and promote its degradation by the 26S proteasome [80]. Upon DNA damage, Mdm2 dissociates from p53 resulting in attenuation of ubiquitylation. The same lysine residues in p53 become acetylated by p300/CBP thus extending the half-life of the p53 protein from approximately 30 min to 5-6 hours [81]. This fact indicates that acetylation and ubiquitination have opposite functional consequences for the protein stability and activity of p53. In a similar way, acetylation of p53 on K320 by PCAF counteracts ubiquitination of the same residue by another E3 ligase, E4F1 [82]. Interestingly, in contrast to ubiquitination of the C-terminus, ubiquitination of the K320 residue does not affect the protein stability of p53, but correlates with its increased binding to the genes involved in the execution of cell cycle arrest program, but not apoptosis [82].

In addition to ubiquitination, p53 can be modified by ubiquitin-like proteins, such as the Small ubiquitin Modifier SUMO. Recently, Wu et al demonstrated that sumoylated p53 failed to undergo acetylation, even though the interaction with p300 was still preserved. This suggests that sumoylation of K386 prevents the subsequent acetylation of C-terminal lysines in p53, possibly contributing to a loss of DNA-binding activity of p53, and thus attenuating p53 transcriptional activity [83].

#### Methylation-acetylation interplay

Besides the acetylation-ubiquitylation competition for the target lysines of p53, there is also interplay between methylation and acetylation. Several studies have demonstrated that Set7/9-mediated methylation of p53-K372 facilitates acetylation of the adjacent lysine residues (K373 and K382) in p53 [59,62]. The exact molecular mechanism of how mono-methylation of K372 activates and stabilises p53 is yet to be discovered. However, Ivanov and colleagues [59] showed that methylation of p53 preceded and enhanced the appearance of acetylation, but not vice versa. The authors proposed that p53-K372 methylation induced it subsequent acetylation by p300/CBP on K373 and K382. Importantly, this enhanced acetylation was evident only within the fraction of p53 bound to chromatin, whereas the pool of unbound nuclear p53 molecules was largely unaffected by Set7/9-mediated methylation.

Interestingly, methylation of p53 on K369 in mice (corresponds to K372 in humans) was also shown to enhance the binding of Tip60 HAT. This enzyme contains a methyl-lysine-binding chromodomain, by which Tip60 recognises methylated K372 residue and promotes acetylation of p53 [62]. However, it is important to note that Tip60 targets only one lysine, K120, which is located in the DNA binding domain of p53 and thus does not explain how K372 methylation enhances the level of acetylation in the C-terminus of p53.

A more plausible explanation to this phenomenon was provided recently in the work of Liu X et al[84] where Set7/9 was found to interact with NAD-dependent HDAC, SIRT1 (Sirtuin 1). This interaction was enhanced by DNA damage stress, correlating with the increased acetylation status of p53 [84]. Interestingly, p53 was also able to interact with SIRT1. However, this interaction was abolished in the presence of Set7/9, thus preventing SIRT1-mediated deacetylation of p53. Collectively, it is likely that Set7/9, by binding Sirt1, imposes a physical hindrance to SIRT1 and thus preserves the high level of acetylation of p53 and its transcriptional activation [84].

#### Lysine methylation interplay

A cross-talk between lysine methylation on different lysine residues in the C terminus of p53 also exists. For example, methylation reactions by Smyd2 on K370 and by Set7/9 on K372 have antagonistic effect on the function of p53: K370me1 repressed p53 and on the contrary, K372me1 activated it. Moreover, K372me1 is induced by DNA damage and prevents Smyd2-mediated methylation of K370, suggesting that lysine methylation of p53 is dynamic [66]. On the other hand, the negative effect of Smyd2 on p53 function may be a result of methylation of histones. Smyd2 is known to interact with two components of the Sin3 histone deacetylase complex, HDAC1 and Sin3A, which could deacetylate histones and thus repress p53-dependent transcription [67].

Collectively, methylation of p53 might be an important step for its subsequent acetylation, which in turn contributes to the increased stability of p53, and enhances its DNA binding and transactivation potential in response to specific cellular stress.

#### Regulation of p63 and p73 by acetylation-ubiquitylation interplay

Besides TP53 itself, there are two other members of the p53 family, TP63 and TP73. Due to their structural similarities with p53, particularly in the DNA binding domain, p63 and p73 are able to bind p53-target promoters and transactivate p53-responsive genes, contributing to cell cycle arrest and/or apoptosis [85-87]. Therefore, perhaps not surprisingly, p53 family members are themselves targets for a variety of PTMs [88].

Similar to p53, the p73 protein is acetylated on the C-terminal lysines (K321, K327 and K331) by p300 upon DNA damage, resulting in transactivation of pro-apoptotic gene, p53AIP1, but not the cell cycle regulating gene, p21 [89-91]. This acetylation was stimulated by the YAP1 protein (Yes-associated protein), which was shown to stimulate the interaction between the N-terminus of p73 and p300 [92]. Furthermore, YAP1 prevents p73 from ubiquitination by the HECT E3 ubiquitin ligase Itch, which interacts with p73 through the same PPPY motif, as YAP1 [93]. Again, similar to p53, SIRT1 decreased the level of p73 acetylation thereby blunting the apoptotic response [94]. p73 can also be mono-ubiquitylated by the E3 Cullin4A (cul4A)-dependent ligase (CDL4A), which does not affect p73 stability, but negatively regulates p73 mediated transcription activity [95].

Collectively, the same regulatory mechanism of competition between acetylation and ubiquitylation demonstrated in p53, is also true for p73, as both its activity and stability are regulated through a physical competition between acetylation by p300 and ubiquitylation by various E3 ubiquitin ligases [96].

Like the two other members of the family, p63 activity can also be regulated by p300. Indeed, acetylation of p63 by p300 led to a specific increase in transcription of the p21 gene, resulting in cell cycle arrest [97]. Also, an alternatively spliced isoform of p63 lacking the N-terminus region (ΔNp63α) was shown to interact with HDACs 1 and 2 in Squamous Cell Carcinoma (SCC) cells, forming a transcriptional repressor complex [98]. The formation of this complex resulted in a decreased transcription of the pro-apoptotic gene PUMA [98], suggesting that p300-mediated acetylation is critical for anti-tumorigenic function of p63. Also, the hinge region of ΔNp63α can be acetylated by PCAF in response to high cellular density. PCAF-mediated acetylation of ΔNp63α resulted in its cytoplasmic sequestration and cell cycle arrest [99].

So far, very little is known about ubiquitylation of p63 [100]. Indeed, only a few E3 ligases have been proposed to be able to ubiquitylate p63. RACK1 (receptor for protein kinase C) was shown to promote ubiquitylation of p63, although it does not contain either HECT or RING-type domains, characteristic of typical E3 ubiquitin ligases [101]. The HECT domain-containing E3 ligase NEDD4 was shown to ubiquitylate and lead to the degradation of ΔNp63α, causing altered dorso-ventral patterning in zebrafish [102]. Another ubiquitin ligase that can also target p73, Itch, was shown to be able to promote ubiquitin-mediated degradation of p63 [103]. This modification seems to be crucial for normal development, as the two lysine residues of p63 that are ubiquitylated by Itch are found mutated in the limb malformation syndrome, split-hand/foot malformation (SHFM).

These results demonstrate the importance of two competing lysine-specific modifications, acetylation and ubiquitylation, in the regulation of the p53 family members. Intriguing questions remain to be answered as whether p63 and/or p73, reminiscent of p53, undergo lysine methylation and how this PTM affects their cellular functions.

#### Parallel with Histone Modifications

Initially, various PTMs were studied in histone proteins due to their abundance. Histones are small, positively charged proteins whose N-termini are rich in lysines and are thus subject to various PTMs. Four pairs of histones (H2A, H2B, H3 and H4) form an octamer on the DNA, called nucleosome. Nucleosomes are considered as the minimal unit of chromatin, which plays an important role in regulation of gene expression [19]. The ‘Histone code’ hypothesis proposed by Allis and Jenuwein [104] suggests the existence of interplay between various post-translational modifications in histone tails. The presence of these multiple post-translational marks would form a unique chemical surface that could be recognized by specific effector molecules, such as transcription factors and/or their co-regulators. Ultimately, this would translate into different functional responses. Such principles could be extended to both histones and non-histone proteins, such as p53, resulting in a more generalized concept of a ‘protein code’ [19,71,105,106].

#### Acetylation

As discussed before, acetylation of p53 correlates with protein stabilization and the activation of p53's transcription response. In a similar way, lysine acetylation of histone proteins is known to decrease the interaction between the histones and the DNA, leading to a more relaxed chromatin state, and thus facilitating the access of the transcriptional machinery to the DNA [107] (Table 1). A large number of lysine acetyltransferases (KATs), including p300/CBP, PCAF and Tip60 acetylate both p53 and histones, suggesting that these events are coordinated.

**Table 1:** KATs involved into regulation of p53 and histones and their functional outcomes

Acetyltransferase	Target residue in Histones	Functional outcome	Target residue in p53	Functional outcome
CBP/p300	H2A/B H3 H4K5,8,12,16	Transcriptional activation	K164, K370, K372, K373, K381, and K382	Stabilisation of p53 Increase p53-dependent transcription
PCAF	H3 H4	Transcriptional activation	K320	p21 induction
Tip60	Free histones H2AK5 H3K14 H4K5,8,12,16	Transcriptional activation Chromatin remodelling	K120	p53-dependent apoptosis
MOF	H4K16	Chromatin remodelling [127]	K120	p53-dependent apoptosis
MOZ	H2B, H3K14 H4K5,8,12,16 (in vitro) H3K9 (in vivo)	Transcriptional activation Chromatin remodelling	K120	p21 induction

#### Methylation

In contrast to acetylation, lysine methylation of histones, much like what occurs in p53, can lead to different responses, either activating or repressing transcription, depending on the location and degree of methylation (mono-, di- or tri-methylation) [108]. Indeed, several enzymes that were identified to methylate p53 were firstly described to methylate lysine residues in histones (Table 2). For example, Set7/9 methyltransferase, which is known to target K372 in p53, was originally described to mono-methylate lysine 4 at histone 3 (H3K4) resulting in transcriptional de-repression [109,110].

**Table 2:** KMTs involved in regulation of p53 and histones and their functional outcomes

Methyltransferase	Target residue in Histones	Functional outcome	Target residue in p53	Functional outcome
SET7/9	H3K4 me1	Transcriptional activation	K372 me1	Up-regulation of p53-target genes
SET8	H4K20 me1	Transcriptional Repression?	K380 me1	Transcriptional inactivation
SMYD2	H3K36 me2	Transcriptional repression	K370 me1	Transcriptional inactivation
G9a/Glp	H3K9 me1 H3K9 me2 H3K27 me1	Gene silencing Heterochromatin formation	K373 me2	Transcriptional Repression?
SMYD3	H3K4 me2	Transcriptional activation	?	?
SETD2	H3K36 me3	Gene regulation?	?	Up-regulation of apoptotic and cell cycle arrest genes
SET1	H3K4 me3	Chromatin Remodelling Transcriptional activation	?	?

Smyd2, is another KMTase that methylates both p53 (K370) and histone H3 (K36) leading to transcriptional repression[67]. Smyd2 has been shown to specifically associate with the Sin3 histone deacetylase complex, particularly with Sin3A and HDAC1[67]. Collectively, these data indicate that Smyd2 is an oncogenic factor that promotes tumorigenesis via inactivation of p53 and repression of its target genes. In line with its oncogenic role, overexpression of Smyd2 has been associated with increased tumour malignancy in esophageal squamous cell carcinoma[111].

Another p53 inhibitory KMTase, Set8, also specifically mono-methylates H4K20[112]. The role of Set8-mediated mono-methylation of H4K20, however, remains elusive. The initial findings showed that meH4K20 was mainly found in euchromatin and seemed to be cell cycle regulated[112-114]. Later studies revealed H4K20 methylation to be associated with heterochromatin regions and thus was linked with transcriptional repression [115,116].

Two KMTases, G9a and Glp, responsible for K373 methylation of p53, also catalyse mono and di-methylation of H3K9 and mono-methylation of H3K27 [117]. Methylation of H3K9 and H3K27 creates binding sites for transcription repressor complexes, such as heterochromatin protein 1 (HP1) and Polycomb (Pc), respectively [118-120]. Much like methylation of K373 in p53, G9a-mediated methylation of H3K9 leads to transcriptional repression [121]. Summarising these data it becomes increasingly apparent that methylation of p53 and histones by a particular KMTase is coordinated and determines the specificity of transcriptional outcome.

**Figure 4:**
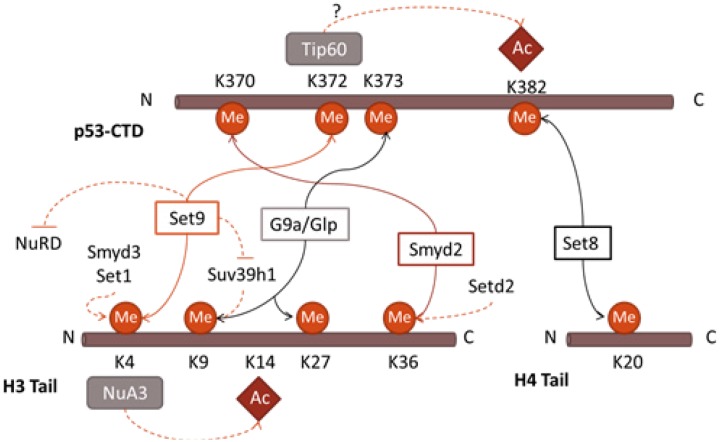
Parallels in interplay between PTMs in p53 and histones. The methyltransferase Set7/9 is able to methylate p53K372 and H3K4, conducting the further acetylation of nearby residues. In addition, it prevents the activity of the deacetylase NuRD complex and methylation of K9H3. Smyd2 and Set8 repress the transcriptional activity of both p53 and histones, mediating the methylation of K370 and K382 in p53, and H3K36 and H4K20 in histones. G9a/Glp mediates methylation of K9 and K27 in H3, and K373 methylation in p53.

Interplay between modifications, in particular methylation/acetylation, seems to occur in both histones and p53 in a similar way. For example, Set7/9 methylates p53 on K372, which augments acetylation of K373 and K382 [59]. Likewise, methylation of H3K4 by Set7/9 was shown to correlate with transcription activation by preventing the interaction of histone H3 with the HDAC complex NuRD, thus hindering deacetylation of H3K9 [109]. Moreover, methylation of H3K4 increased subsequent acetylation of H3K14[109,122](Figure 4).

The regulation of p53 by methylation and acetylation closely resembles the one uncovered in histones, with both sharing similarities at many levels. Thus, by analogy, it is tempting to extrapolate the existence of other PTMs in histones, previously found in p53. If true, then various E3 ubiquitin ligases that interact with p53, should also ubiquitinylate histones. Similarly, a large cohort of kinases that bind p53 should phosphorylate histones as well. Future experiments should test the validity of our hypothesis.

The opposite situation might also be true, i.e. specific KMTs that methylate histones in various positions within chromatin of the p53-dependent promoters may also modify p53 itself. Strikingly, the sequence similarity between the H4 tail around K20 and p53 at K382 was predictive not only of Set8 as a major KMT that methylates these residues, but also of two interacting effector proteins subsequently identified: L3MBTL1 and 53BP1 [71].

By the same token, promoters of many p53-dependent genes contain elevated levels of H3-K4 tri-methylation, which is executed by the MLL (COMPASS) complex. Thus, not surprisingly, MLL1 was shown to interact with p53[123]. The question remains to be answered is whether MLL1 methylates p53 itself. On the related note, Suv39-h1 implicated in H3K9 methylation and regulation of p53-target promoters, exhibits weak but specific methylation activity towards p53 (N.B. unpublished results) [124].

#### Lysine modification by metabolites

It has been shown recently that a reactive intermediate formed during glycolysis, 1,3-bisphosphoglycerate, can non-enzymatically modify specific lysines in proteins, providing a mean by which accumulation of metabolic intermediates exerts a regulatory feedback to control flux through various pathways. Proteomic analysis showed that the 3-phosphoglyceryl-lysine (3-pgK) is formed upon interaction of particular lysine residues with 1,3-bisphosphoglycerate. This PTM was produced naturally in cells and was enriched in proteins that function in glycolysis. In addition to glycolytic enzymes, several modulators of p53 function were identified to undergo this modification, i.e. Sirt1 and 14-3-3 [125].

Importantly, p53 influences metabolic pathways by regulating the levels of a series of gene products that affect metabolic fates and metabolic products. For example, p53 increases the expression of synthesis of cytochrome c oxidase 2 (SCO2), which blocks the hexokinase pathway of conversion required for the production of 1,3-bisphosphoglycerate [126]. Thus, p53 may indirectly regulate the level of this PTM and hence affect the activity of glycolytic and nuclear enzymes. It remains to be seen whether p53 by itself undergoes non-enzymatic 3-pgK modification in tumours under hypoxic conditions.

## FINAL REMARKS

Post-translational modifications represent, incontestably, a very complex regulatory mechanism that allows the cell to tightly control the functions of various proteins. The regulation of the tumour suppressor p53 by post-translational modifications provides an additional layer of control that can dictate its biological response. Indeed, post-translational modifications can produce a multitude of outcomes on p53, altering its transcriptional activity. The comprehension of the exact consequences of posttranslational modifications in both p53 and histones would help defining the response of p53 to specific anti-cancer therapy. To address this question it is important to know the enzymes responsible for the particular PTM as well as the effector molecule that “reads” this modification and defines specific functional outcome. In this respect, we propose an easily testable concept whereby using the index of post-translational modification patterns in histones one should be able to define a predictable biological response of some post-translational modification patterns found in p53 in response to various forms of stress.
